# Correlates of physical activity behavior in adults: a data mining approach

**DOI:** 10.1186/s12966-020-00996-7

**Published:** 2020-07-23

**Authors:** Vahid Farrahi, Maisa Niemelä, Mikko Kärmeniemi, Soile Puhakka, Maarit Kangas, Raija Korpelainen, Timo Jämsä

**Affiliations:** 1 Research Unit of Medical Imaging, Physics and Technology, University of Oulu, P.O. 5000, FI-90014 Oulu, Finland; 2 Medical Research Center, Oulu University Hospital and University of Oulu, Oulu, Finland; 3 Center for Life Course Health Research, University of Oulu, Oulu, Finland; 4 Department of Sports and Exercise Medicine, Oulu Deaconess Institute Foundation sr, Oulu, Finland; 5 Geography Research Unit, University of Oulu, Oulu, Finland; 6 Diagnostic Radiology, Oulu University Hospital, Oulu, Finland

**Keywords:** Decision tree, CHAID, Multilevel model, Prediction, Classification

## Abstract

**Purpose:**

A data mining approach was applied to establish a multilevel hierarchy predicting physical activity (PA) behavior, and to methodologically identify the correlates of PA behavior.

**Methods:**

Cross-sectional data from the population-based Northern Finland Birth Cohort 1966 study, collected in the most recent follow-up at age 46, were used to create a hierarchy using the chi-square automatic interaction detection (CHAID) decision tree technique for predicting PA behavior. PA behavior is defined as *active* or *inactive* based on machine-learned activity profiles, which were previously created through a multidimensional (clustering) approach on continuous accelerometer-measured activity intensities in one week. The input variables (predictors) used for decision tree fitting consisted of individual, demographical, psychological, behavioral, environmental, and physical factors. Using generalized linear mixed models, we also analyzed how factors emerging from the model were associated with three PA metrics, including daily time (minutes per day) in sedentary (SED), light PA (LPA), and moderate-to-vigorous PA (MVPA), to assure the relative importance of methodologically identified factors.

**Results:**

Of the 4582 participants with valid accelerometer data at the latest follow-up, 2701 and 1881 had active and inactive profiles, respectively. We used a total of 168 factors as input variables to classify these two PA behaviors. Out of these 168 factors, the decision tree selected 36 factors of different domains from which 54 subgroups of participants were formed. The emerging factors from the model explained minutes per day in SED, LPA, and/or MVPA, including body fat percentage (SED: B = 26.5, LPA: B = − 16.1, and MVPA: B = − 11.7), normalized heart rate recovery 60 s after exercise (SED: B = -16.1, LPA: B = 9.9, and MVPA: B = 9.6), average weekday total sitting time (SED: B = 34.1, LPA: B = -25.3, and MVPA: B = -5.8), and extravagance score (SED: B = 6.3 and LPA: B = − 3.7).

**Conclusions:**

Using data mining, we established a data-driven model composed of 36 different factors of relative importance from empirical data. This model may be used to identify subgroups for multilevel intervention allocation and design. Additionally, this study methodologically discovered an extensive set of factors that can be a basis for additional hypothesis testing in PA correlates research.

## Introduction

The positive relationship between physical activity (PA) and health has been well established [1, 2], yet many adults worldwide perform insufficient PA [3]. Thus, understanding the factors associated with PA behavior is essential to develop and improve public health interventions [3–5]. Many studies have investigated the association of various factors including personal, societal, and environmental factors with different PA behavior indices such as the daily amount of moderate-to-vigorous PA (MVPA) or sedentariness [5–7]. Despite much progress in research into correlates, only a few studies have followed analytical approaches that account for both the existence of several levels of influence [5, 7, 8] and the complexity and multimodality of PA behavior [5, 9, 10]. To further advance correlates research, there has been calls for more research using both sophisticated statistical assessment that can capture the multilevel nature of correlates [4, 5] and PA behavior definitions that better reflect everyday life rather than unidimensional metrics such as daily MVPA [1, 5, 9, 10].

Using classical statistical modeling (such as regression analyses), studies have generally examined whether and how various factors are associated with different PA metrics [6, 11]. In classical statistics, these analyses could remain restricted to data analysts’ decisions about how the association and interaction are hypothesized (knowledge-driven) mainly because the factors selected for inclusion in the analyses are primarily chosen subjectively according to their conceptual relevance and, in some cases, initial empirical associations [11, 12]. This may limit the recognition of new and innovative correlate categories, which are needed in this field for further progress [5, 11]. Ecological approaches that integrate ideas from several theories have been also used in correlates research, often to overcome classical statistical analysis limitations [5]. They have been used to both conceptualize the factors and their interrelationships at all levels explaining PA behavior (such as the interconnections between individuals and their social and physical environments) [13] and guide variable selection for analyses [5, 11]. However, ecological approaches are also knowledge-driven [6] and, to some extent, rely on very well-established correlates [6, 8], which might result in missing some factors and interrelationships associated with PA behavior.

We have now entered a data-intensive era, with an increasing popularity of data mining approaches [14]. Such approaches originated from statistics but are known to capture hidden and novel insights buried in large amounts of data and generate data-driven hypotheses [14, 15]. These principles also regard the field of PA research, in which there is a need for more complex approaches to identify the next generation of PA behavior correlates, understand their relative importance, and capture the complex interrelations among the factors at different levels [5, 6, 8]. Several studies have applied data mining approaches [16–19] mostly to establish data-driven correlate hierarchies [16, 17] but using a limited number of factors and self-reported measurement of PA or sedentary behavior.

The present study applied a predictive data mining approach to classify individuals’ PA behavior (defined as active or inactive) using an extensive list of individual, demographical, psychological, behavioral, environmental, and physical factors. PA behavior, to better represent everyday life, was defined based on machine-learned activity profiles established preciously using a multidimensional (clustering) approach applied on continuous accelerometer-measured activity intensities in one week [20]. This cross-sectional study sought to build a data-driven hierarchy of PA behavior correlates from empirical data and, as a secondary purpose, to methodologically identify PA behavior correlates from a wide list of factors.

## Materials and methods

**Fig. 1:**
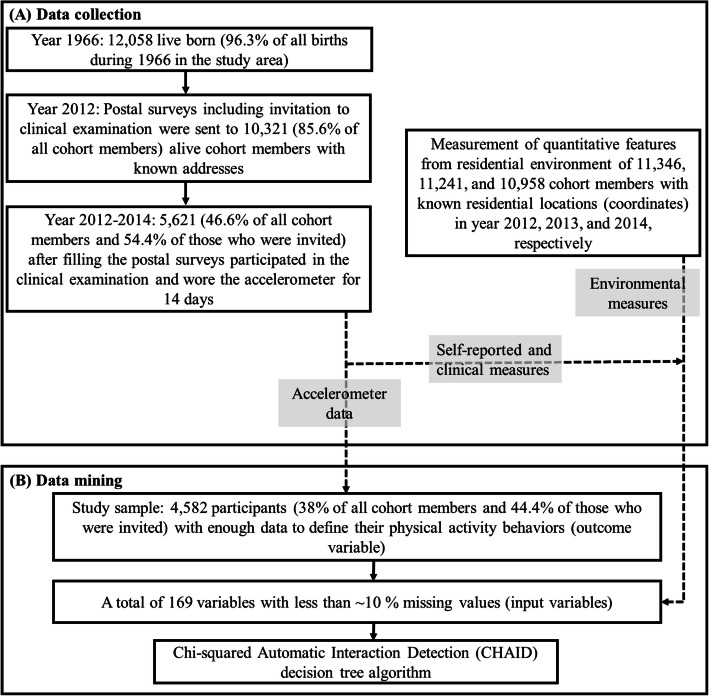
The collected data in the latest follow–up of Northern Finland Birth Cohort 1966 (**a**), and the selection of study population, input variables, and outcome variables for data mining in the present study (**b**)

Data for the present study were from the population-based Northern Finland Birth Cohort 1966 study (NFBC1966). NFBC1966 is a life-course study involving participants whose dates of birth were expected to be in 1966 in Finland’s two northernmost provinces, Oulu and Lapland (*n* = 12,058, 96.3% of all live births in the study area). The present cross-sectional study included NFBC1966 cohort members who participated in the latest follow-up at age 46 and agreed to wear accelerometers for device-based physical activity measurements [21]. A total of 10,321 NFBC1966 cohort members (85.6% of all cohort members) were alive in Finland in 2012 and were invited to the follow-up, of which 5621 (46.6% of all cohort members and 54.4% of those who were invited) participated and wore accelerometers (Fig. 1). With respect to the measurement tools/techniques, the collected data can be categorized into four: self-reported measures, clinical measures, objective built and natural environmental measures, and objective physical activity measures.

### Questionnaires and measurements

#### Questionnaires

A postal questionnaire was sent to all living cohort members with known addresses. The questionnaire included items on social background, frequency and type of habitual exercises, physical and psychological health and well-being, and work–life and socioeconomic situation. In addition, health-related behaviors were assessed by a separate questionnaire, the Quality Of Life Questionnaire (15D©), to rate health-related quality of life [22]. Another additional separate survey was used to address opinions and experiences, covering questions from the Temperament and Character Inventory (TCI) questionnaire [23]. The temperament and personality trait scores were then composed based on the responses to the items of the TCI questionnaire. More details on the self-reported measures can be found elsewhere [24].

#### Clinical examination and measurement of physical activity

Participants were also invited to attend a clinical examination. The clinical examinations included measurement of anthropometry, body composition, and cardiorespiratory fitness. Participants’ height, weight, blood pressure and waist-hip ratio were measured and BMI (body mass index) calculated. Participants’ body composition was measured with bio-impedance measurement (InBody720, InBody, Seoul, Korea). A static back muscle strength test (Biering-Sorensen trunk extension test) was performed to evaluate physical performance. A submaximal four-minute single-step test during which heart rate was continuously monitored was performed to assess cardiorespiratory fitness. Further details on the clinical examination protocol and measures are presented elsewhere [25, 26].

Objective measurement of physical activity was initiated during clinical examination using a wrist-worn accelerometer (Polar Active, Polar Electro Oy, Kempele, Finland). Participants were instructed to wear the monitor on the wrist of their non-dominant hand continuously for 24 h for 14 days. Polar Active has a uniaxial accelerometer that outputs estimated energy expenditure in metabolic equivalent (MET) values every 30 s. The validity of Polar Active under free-living conditions against the double-labeled water technique has been shown elsewhere [27].

#### Environmental measures

We obtained the residential coordinates of all participants whose residences were available at the time of the 46-year follow-up data collection (2012–2014) from the Finnish Population Register Centre. We used a geographic information system (ArcGIS 10.3) to calculate built, natural, and socioeconomic environment variables (Supplementary file 1, Table S1) that might describe the conduciveness of participants’ residential environment to PA. We calculated all variables in the year the participant attended the 46-year data collection. We also determined quantitative environmental features using a one-kilometer-radius circular buffer around the residential locations, and the distances (as the crow flies) to amenities were measured using road network data.

Data related to community structure; land use; amenities such as retail, recreation, office, and community institutions; and socioeconomic factors were derived from the Finnish community structure database [28]. Street network data, including the number of bus stops, intersection density, and length of cycle paths, were based on the Finnish national road and street database (Digiroad) [29]. Data on indoor and outdoor sport facilities were obtained from the Finnish database of sport facilities [30]. Natural environment features such as distances to the closest forests and parks and residential area greenness were assessed with the land cover data from the Finnish Environment Institute [31].

### Data mining using a decision tree

We selected a decision tree technique to establish a data-driven model for classifying PA behavior. A decision tree model is created by partitioning the data on the basis of several independent input variables (or predictors) to form homogenous subgroups with respect to the outcome variable. A decision tree-produced hierarchy has a flow chart-like structure that enables identifying the relative importance of input variables in predicting the outcomes; the predictors in the higher layers of hierarchy are more important predictors [32]. In clinical applications and several other areas in which interpreting the results is of vital importance, decision trees are one of the most widely used classification methods [12, 14, 32, 33].

We used the Chi-squared Automatic Interaction Detection (CHAID) decision tree algorithm to create the model [34]. CHAID has been repeatedly used in studies with clinical applications whose main purpose was to identify key factors related to the outcomes of interest [35, 36]. In this algorithm, homogenous groups may be formed by any possible combination of the known values of a categorical predictor, or by setting cut-off points at any values of a continuous predictor. The number of selected independent predictors for creating the model together with the number categories (for categorical and ordinal) and intervals (for continuous) for the selected independent predictors depends on results of the Chi-square analyses and whether the differences are significant or not. Since the correlates of PA behavior could be of mixed data types, CHAID is an appropriate candidate because it uses a nonparametric procedure with no assumptions of the underlying data and is designed to include continuous, ordinal, and categorical predictors [33].

### Decision tree model construction and validation

#### Input variables (predictors) and physical activity behavior (outcome variable)

The questionnaire and clinical and environmental measures, except those with more than ~ 10% missing values, were used as input variables. Recent evidence suggests that any single unidimensional metric (including the most commonly used criterion that defines physical inactivity as the insufficient activity level to meet present recommendations [1]) might not be enough to define individuals’ PA behavior [10, 37–39]. We therefore used participants’ activity profiles, which we built in a previous study using a multidimensional approach and continuous accelerometer data to define the PA behaviors for the present study [20]. A distinct aspect of this approach is that continuous accelerometer-measured activity intensities in one full week across the whole intensity continuum, including sedentary (SED), light PA (LPA), and MVPA were incorporated into a machine learning approach to create the activity profiles.

The details about how the activity profiles were established have been presented elsewhere [20]. Briefly, X-means clustering algorithm was applied on accelerometer-based MET-level data of participants who had seven consecutive valid measurement days (*N* = 4582), and four distinct activity profiles (clusters) were derived. A total of 1008 features/variables (10-min averages of the original 30-s MET data resulting in 144 MET values for each of the 7 valid measurement days) for each participant were fed into the clustering algorithm for creating the profiles [20]. A valid measurement day was defined as at least 600 min of activity monitor wearing time per day during waking hours. Seven consecutive valid measurement days were used as a criterion to enable analyzing one full week including both weekdays and weekends. The activity profiles were named with respect to the temporal and intensity patterns of participants’ daily activities in each cluster: Inactive (*N* = 1881), Moderately active (*N* = 802), Evening active (*N* = 1297), and Very active (*N* = 602). The results of our initial experiments revealed the decision trees induced for classifying the four activity clusters have unreasonable performance and generalizability, primarily because the outcome variable had both class imbalance (i.e., 41% Inactive, 18% Moderately active, 28% Evening active, and 13% Very active) and class overlap (i.e., those who were in the Moderately active, Evening active, and Very active had comparable activity profiles with different temporal patterns) problems [40]. Previous research has shown that the effects of these two problems that associate with each other in limiting the performance and generalizability of classification trees is best minimized with near-balanced class distribution in the outcome variable [41]. We therefore defined those in the Moderately active, Evening active, or Very active clusters as *active* (*N* = 2701), and the remaining ones who were in the Inactive cluster as *inactive* (*N* = 1881). We used the input variables in their original form to classify the two PA behavior categories: *active* and *inactive*.

#### Missing values and algorithm parameters

Missing values were included in the analysis as a separate category that was allowed to merge with other categories in the decision tree. The imputation of missing values of input variables was unnecessary [35]. A previous study has shown that the a decision tree developed with the presence of missing values in their input variables has reasonable misclassification rates, especially when the missing values are not very high (e.g., 20%) [42].

Several parameters must be set prior to constructing a decision tree model. Of these parameters, pruning criteria are the most primary ones to limit the size of the tree and prevent overfitting [14]. The pruning criteria were set such that groups smaller than 80 were not split any further (maximum number of participants in a parent node), and no group smaller than 40 was formed (maximum number of participants in a child node). The tree growth was limited to 10 layers, meaning that a maximum of 10 factors could be selected to form a group.

#### Model validation and visualization

We created and validated the model using 10-fold cross-validation. To evaluate the accuracy of the final decision tree model, we used the confusion matrix, which shows the proportion of participants with each outcome variable that was correctly and incorrectly classified. In the visualization of the final tree, the percentage of active and inactive participants in each subgroup, along with the response index (RI), was presented. The RI is the percentage of inactive participants in each subgroup relative to that of inactive participants in the total sample (i.e., 41.1%). Similar to an odds ratio, RI is an indicator of the direction and strength of the association [16].

### Activity patterns in decision tree-formed subgroups of participants

Given that the outcome variable was formed with a multidimensional approach, we also calculated Z-scores of three PA metrics including average daily time (minutes per day [min/day]) spent in SED, LPA, and MVPA in each decision tree-formed subgroup of participants. A Z-score indicates how many standard deviations the mean of a measure in a subgroup is away from the corresponding mean in whole study population. As such, we could compare the variation of the three activity intensities across different subgroups with respect to the study population means. We calculated these three PA metrics from the same seven consecutive valid measurement days to establish the activity profiles [20] using previously validated cut-points (SED, 1–1.99 MET; LPA, 2–3.49 MET; and MVPA, ≥ 3.5 MET) by the accelerometer manufacturer [43].

### Association analysis

The same above-mentioned PA metrics (SED, LPA, and MVPA) were also used for association analyses. We examined the association between factors emerging from the model and these PA metrics to determine the significance and relative importance of the methodologically identified factors. We used adjusted generalized linear mixed models, including urban–rural area as a random effect, to examine the associations between each independent variable (factor emerging in the decision tree) separately with min/day in SED, LPA, and MVPA. Age and gender were used as covariates in all models. We standardized the continuous independent variables to obtain a mean of zero and a standard deviation (SD) of 1 before including them in regression analyses. As such, we could interpret coefficients (B) from the models encompassing a continuous independent variable as a change in the outcome (e.g., min/day of LPA) for every 1 SD change in the independent variable and therefore compare them to each other across a similar outcome in terms of magnitude regardless of the unit. We included the categorical and ordinal independent variables in the regression analyses in the form of dummy variables and set response categories at the lowest end as the reference category. A *p*-value of 0.05 was used to interpret significance. All analyses (including data mining) were performed with IBM SPSS Statistics for Windows, version 25.0 (IBM Corporation, Armonk, USA).

## Results

### Participants

A total of 4582 participants (38% of all cohort members and 44.4% of those invited to the 46-year follow-up) had enough valid PA data to be included in the cluster analysis study [20] and, accordingly, sufficient information on the outcome value (active or inactive profile) for inclusion in the present study. The numbers of participants with an active and inactive profiles were 2701 (58.9%) and 1881 (41.1%), respectively. The characteristics of the study’s participants for the whole sample, with respect to the two outcome variables, are shown in Table 1. These descriptive results are identical to those reported in cluster analysis study [20].

**Table 1:** The characteristics of the study participants

	Inactive profile (***n*** = 1881)	Active profile (***n*** = 2701)	Total population (***n*** = 4582)
Height, cm (SD)	168.4 (8.9)	172 (9.0)	170.5 (9.1)
Weight, kg (SD)	77.3 (17.1)	78.6 (16.2)	78.1 (16.6)
Body mass index, kg/m^2^ (SD)	27.2 (5.2)	26.4 (4.5)	26.7 (4.8)
Body fat, % (SD)	31.2 (9.3)	27.7 (8.9)	28.9 (9.2)
Alcohol consumption, grams/day (SD)	9.8 (4.1)	10.4 (16.8)	10.1 (4.3)
Gender			
Men	648 (34)	1268 (44)	1916 (42)
Women	1233 (66)	1433 (45)	2666 (58)
Education			
No professional education	53 (3)	85 (3)	138 (3)
Vocational/college level education	1119 (60)	1765 (65)	2884 (67)
Polytechnic/university degree	573 (30)	670 (25)	1243 (29)
Employment status			
Employed	1499 (78)	2265 (84)	3764 (88)
Student	38 (2)	32 (1)	70 (2)
Unemployed	117 (6)	101 (4)	218 (5)
Other	95 (5)	88 (3)	183 (4)
Marital status			
Married/cohabiting	1421 (75)	2072 (77)	3493 (86)
Divorced	182 (10)	229 (8)	411 (10)
Unmarried	192 (11)	272 (10)	464 (11)
Widowed	11 (0.5)	6 (0.2)	17 (0.4)
Smoking			
Non-smoker	990 (55)	1413 (55)	2403 (55)
Current smoker	335 (19)	447 (18)	782 (18)
Former smoker	458 (26)	702 (27)	1160 (27)
SED, min/day (SD)	675.1 (78.9)	608.2 (91.7)	635.6 (92.7)
LPA, min/day (SD)	242.4 (59.3)	309.8 (71)	282.1 (74.3)
MVPA, min/day (SD)	48.3 (20.6)	83.8 (36.6)	69.2 (33.6)

Values are numbers (%) if not otherwise stated. *SD* Standard deviation, *SED* Sedentary, *LPA* Light physical activity, *MVPA* Moderate-to-vigorous physical activity

### Input variables

We used a total of 168 factors as input variables after eliminating those with over ~ 10% missing values. Overall, the factors related to medication use and diseases had the highest number of missing values (~ 20–50%) while the number of missing values in environmental and adiposity-related factors were lowest (~ 1–5%). Of these 168 factors, 82 were continuous, 19 were categorical, and 67 were ordinal factors. All the 168 input variables are given in the Supplementary file 1, Tables S1–S3.

### Decision tree model

**Fig. 2:**
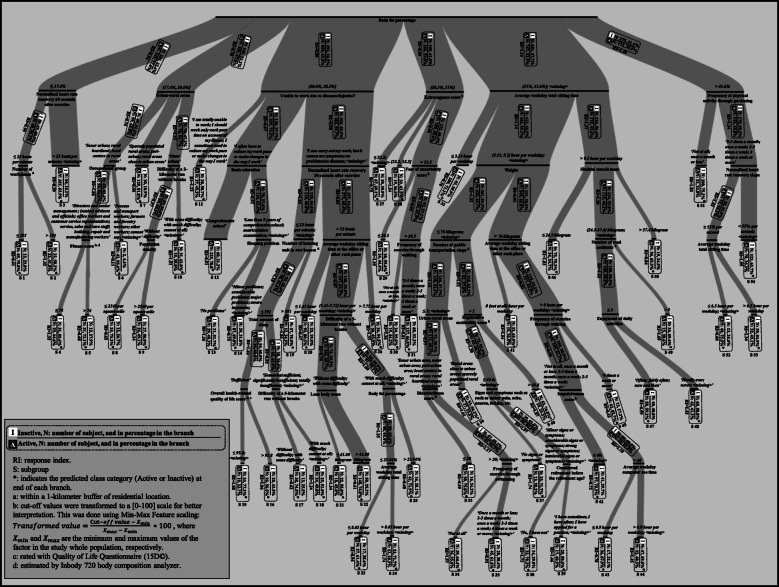
The Chi Squared Automatic Interaction Detection tree illustrating the hierarchy of the factors predicting Active and Inactive participants. The thickness of branches is based on the number of participants in the branch. Categories (for categorical and ordinal variables) and cut-off values (for continuous variables) are shown in italicized text, and the variables in normal text. In interval notations between brackets, inclusiveness and exclusiveness are shown with squared and round brackets, respectively

The prediction results are presented in Table 2. The overall classification accuracy was 69.7%. The final decision tree is shown in Fig. 2. The decision tree algorithm selected a total of 36 different factors of different domains, by which 54 subgroups of participants were formed (marked in Fig. 2 as S1-S54), 26 predicted as active and 28 as inactive. The most frequently appeared factor in the model, appearing three times, was ‘average weekday total sitting time’, followed by ‘average weekday sitting time at the office or such places’, ‘body fat percentage’, ‘frequency of exercise through walking’, ‘urban-rural areas’, and ‘difficulty of a 5-kilometer run without breaks’, which each appeared twice. Other variables appeared only once. The number of layers (or factors) for forming subgroups ranged from two to seven, even though the allowed maximum number of layers was 10.

**Table 2:** Confusion matrix showing the performance of model with 10-fold cross validation

	*Predicted outcome*		Percent correct
*Actual outcome*	Active, n	Inactive, n	
Active, n	2014	687	**74.6%**
Inactive, n	705	1176	**62.5%**

Overall, participants with higher body fat percentage (> 31%) were more likely to be inactive (RI range: 1.16–1.49) compared with those with lower body fat percentage (< 28.3%). The largest subgroup of inactive participants (*n* = 193, RI = 1.55) included those with the highest body fat percentage who reported their physical activity frequency through gardening more than once a month, and were with a normalized heart rate recovery slope < 55% per second. The largest active subgroup (*n* = 335, RI = 0.39) was composed of participants with the lowest body fat percentage in the study population and with a normalized heart rate recovery 60 s after exercise > 25 beats per minute. Participants who lived in city/rural centers and had a physically demanding occupation (i.e., process and transport workers, forestry workers and farmers, and other workers) had the least risk of being inactive (RI = 0.11).

### SED, LPA, and MVPA variations in the decision tree-formed subgroups of participants

**Fig. 3:**
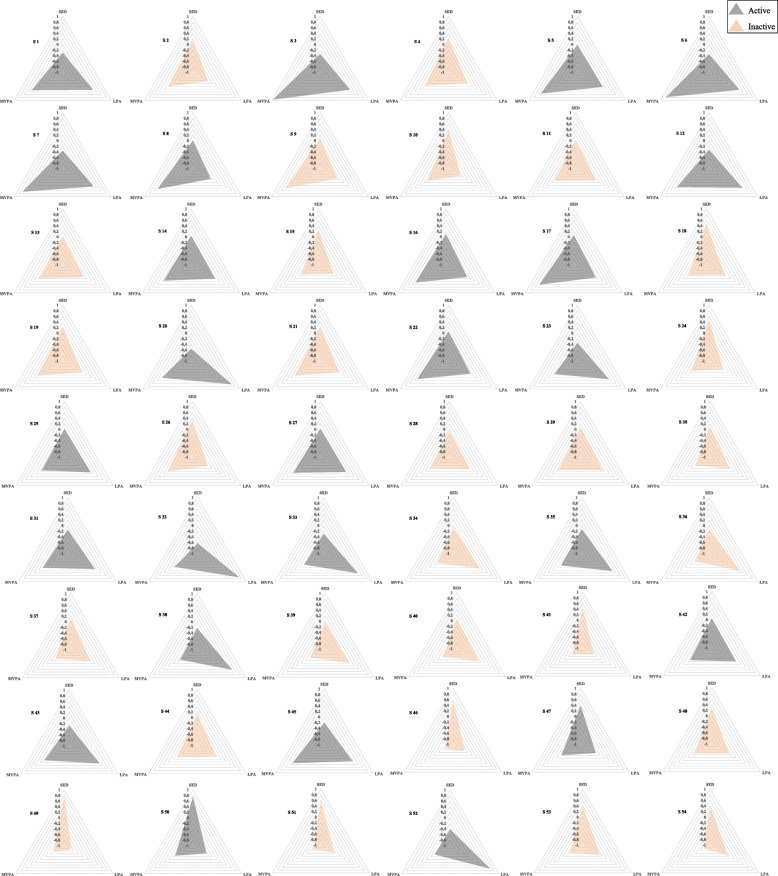
Z-scores of sedentary (SED), light physical activity (LPA), and moderate-to-vigorous physical activity (MVPA) in the 54 decision tree-formed subgroups of participants. S _=_ Subgroup

The variations in the three activity intensities in the 54 decision tree-formed subgroups of participants are shown in Fig. 3. Most inactive and active subgroups had different accumulation patterns of SED, LPA, and MVPA. In general, although most active subgroups had lower SED level than the population mean, some subgroups had noticeably higher levels of MVPA (e.g., subgroups 3, 6, and 7), while others had noticeably higher levels of LPA (e.g., subgroups 20, 32, 33, and 52). Inactive subgroups had generally higher SED level and lower MVPA level than the population mean, while several subgroups had noticeably both lower LPA and MVPA levels (e.g., subgroups 41, 46, 49, and 51).

### Association analysis

Tables 3 and 4 show the association between the continuous, categorical, and ordinal explanatory variables from the decision tree model and the three PA metrics in the total study population. All factors except fear of uncertainty and impulsiveness scores were associated with at least one PA metric. Most continuous factors (Table 3) in the relatively high layers of the decision tree model and larger subgroups significantly explained min/day in all the three PA metrics. For example, body fat percentage was positively associated with SED level (B = 26.5) and inversely associated with LPA (B = -16.1) and MVPA (B = -11.7) levels. Higher normalized heart rate recovery 60 s after exercise was associated with lower SED (B = -16.1) and higher LPA (B = 9.9) and MVPA (B = 9.6). Categorical factors were also associated with min/day in SED, LPA, and/or MVPA (Table 4). For instance, those with physically strenuous occupations (workers, farmers, service, sales, and care staff compared with managers, advisers, office workers, etc.) spent less time in SED (B = -46.7) and more time in LPA (B = 41.1) and MVPA (B = 3.5). Those who reported a higher frequency of physical activity through gardening (2–3 times a month or higher compared with fewer than once month or not at all) had lower SED (B = -20.6) and higher LPA (B = 14.4).

**Table 3:** Associations with the whole study population (N = 4582) between the continuous factors emerged in the decision tree model and time spent in sedenteriness (SED), light physical activity (LPA), and moderate-to-vigorous physical activity (MVPA)

Continuous factors emerged in the decision tree model	Missing values (n)	SED (min/day)		LPA (min/day)		MVPA (min/day)	
		B (95% CI)	p	B (95% CI)	p	B (95% CI)	p
Body fat percentage	75	26.5 (23.5, 29.6)	< 0.001**	-16.1 (−18.5, − 13.6)	< 0.001**	-11.7 (− 12.9, − 10.6)	< 0.001**
Normalized heart rate recovery 60 s after exercise	421	-16.1 (− 18.1, − 13.4)	< 0.001**	9.9 (7.7, 12.1)	< 0.001**	9.6 (8.6, 10.6)	< 0.001**
Extravagance score	336	6.3 (3.5, 9)	< 0.001**	-3.7 (−5.9, − 1.50)	0.001**	−0.6 (− 1.6, 0.5)	0.273
Average weekday total sitting time	204	34.1 (31.5, 36.7)	< 0.001**	− 25.3 (− 27.4, −23.3)	< 0.001**	− 5.8 (− 6.8, − 4.7)	< 0.001**
Number of workplaces	10	2.9 (− 0.1, 6)	0.058	−3.2 (− 5.6, − 0.7)	0.011*	0.6 (− 0.5, 1.7)	0.302
Normalized heart rate recovery 30 s after exercise	399	−16.9 (− 19.6, − 14.2)	< 0.001**	11 (8.8, 13.2)	< 0.001**	9.1 (8 to 10.1)	< 0.001**
Fear of uncertainty score	336	−1.8 (− 4.6, 0.9)	0.198	0.7 (− 1.6, 2.9)	0.553	−0.6 (− 1.7, 0.4)	0.235
Weight	4	13.3 (10.3, 16.3)	< 0.001**	−8.4 (−10.7, − 6)	< 0.001**	−3.4 (− 4.5, − 2.2)	< 0.001**
Skeletal muscle mass	75	− 8.4 (− 13.1, − 3.8)	< 0.001**	3.5 (− 0.1, 7.2)	0.060	9.6 (7.8, 11.3)	< 0.001**
Normalized heart rate recovery slope	425	−17.7 (− 15, − 20.4)	< 0.001**	10.6 (12.8, 8.4)	< 0.001**	9.5 (10.5, 8.4)	< 0.001**
Fitness score	75	−23.4 (− 26.2, − 20.7)	< 0.001**	15.1 (12.9, 17.3)	< 0.001**	10.7 (9.7, 11.8)	< 0.001**
Population density	12	7.5 (3.7, 11.4)	< 0.001**	−7 (−10.1, − 3.1)	< 0.001**	0.8 (− 0.5, 2.2)	0.227
Number of housing unit in row houses	12	3.2 (0.2, 6.2)	0.036*	−2.9 (− 5.3, −0.5)	0.018*	−0.2 (−1.3, 0.9)	0.703
Average weekday sitting time at the office or other such place	382	27.9 (25.2, 30.6)	< 0.001**	−22.4 (− 24.6, − 20.3)	< 0.001**	−2.6 (− 3.6, − 1.5)	< 0.001**
Number of public transportation stops	10	4.1 (1.8, 8.1)	0.002**	−3.6 (− 6.1, − 1)	0.006**	0.3 (− 0.8, 1.5)	0.572
Number of road accidents	10	5.9 (2.5, 9.4)	0.001**	−3.8 (− 6.6, −1.1)	0.006**	0.1 (−1.13, 1.3)	0.876
Explorative excitability score	336	3.1 (0.4, 5.9)	0.026*	−2.2 (− 4.4, 0.03)	0.054	0.6 (−0.4, 1.7)	0.248
Overall health-related quality of life score	334	−8.4 (−11.1, − 5.7)	< 0.001**	5.81 (3.6, 7.9)	< 0.001**	4.1 (3.1, 5.1)	< 0.001**
Lean body mass	75	−8.8 (−13.4, −4.3)	< 0.001**	3.1 (0.3, 7.6)	0.033*	9.4 (7.8, 11.2)	< 0.001**
Disorderliness score	337	6.4 (3.6, 9.1)	< 0.001**	−4.8 (−6.1, −2.6)	< 0.001**	−0.7 (−1.8, 0.3)	0.188
Impulsiveness score	336	2.2 (−0.5, 4.1)	0.112	−1.5 (−3.7, 0.7)	0.176	−0.4 (− 1.4, 0.6)	0.436
Average weekday computer use time	456	14.7 (11.9, 17.5)	< 0.001**	−11.4 (− 13.7, −9.2)	< 0.001**	−4.5 (− 5.5, − 3.4)	< 0.001**

The regression coefficients (B) with (95% confidence interval) from generalized linear mixed model controlling for gender and age with urban-rural area as a random effect. ⁎*p* < 0.05; **p < 0.01

**Table 4:** Associations with the whole study population (*N* = 4582) between the categorical and ordinal factors emerged in the decision tree model and time spent in sedenteriness (SED), light physical activity (LPA), and moderate-to-vigorous physical activity (MVPA)

Categorical and ordinal factors in the decision tree model	Missing values (n)	SED (min/day)		LPA (min/day)		MVPA (min/day)	
		B (95% CI)	p	B (95% CI)	p	B (95% CI)	p
Urban-rural areas (rural area)	10	−20.9 (−40.3, −1.4)	0.35	21.7 (5.3, 38.2)	0.009**	0.2 (−4.7, 5.1)	0.921
Unable to work due to diseases/injuries (no problems/no diseases *or* I can carry out my work, but it causes me symptoms)	209	6 (−1, 13.1)	0.093	−1.4 (−7, 4.1)	0.618	4.4 (1.8, 7.1)	0.001**
Frequency of physical activity through gardening (2–3 times a month *or* more)	207	−20.6 (−27.3, −14)	< 0.001**	14.4 (9.1, 19.6)	< 0.001**	1.4 (−1, 3.9)	0.254
Occupational group (workers, farmers, service, sales and care staff)	261	−46.7 (−52, − 41.3)	< 0.001**	41.1 (36.9, 45.4)	< 0.001**	3.5 (1.4, 5.6)	0.001**
Difficulty of a 2-km run without breaks (without difficulty *or* with some difficulty)	211	−22.9 (− 28.7, − 17.1)	< 0.001**	8.9 (4.3, 13.5)	< 0.001**	14.2 (12.1, 16.4)	< 0.001**
Basic education (less than 9 years of comprehensive school *or* comprehensive school)	206	−17.9 (− 23.6, − 12.2)	< 0.001**	19.9 (15.5, 24.4)	< 0.001**	−2.1 (− 4.2, 0.1)	0.051
Sleeping problems (no problems *or* minor problems)	186	−20.6 (−30.3, − 10.9)	< 0.001**	14.6 (6.9, 22.2)	< 0.001**	5.3 (7.71, 8.9)	0.004**
Frequency of exercise through walking (2–3 times a month *or* more)	195	−16.9 (−24.7, −9.2)	< 0.001**	−3.89 (− 2.2, 10)	.214	9.4 (6.5, 12.3)	< 0.001**
Total amount of sleep (Sufficient *or* somewhat sufficient)	268	−10.1 (− 19.9, −0.4)	0.041*	−0.6 (−8.3, 7.2)	0.88	2.5 (− 1.1, 6.2)	0.176
Difficulty of a 5-km run without breaks (without difficulty *or* with some difficulty)	201	−24.2 (− 29.8, − 18.7)	< 0.001**	8.3 (3.8, 12.7)	< 0.001**	16.1 (14, 18.1)	< 0.001**
Enjoyment of daily activities (often *or* fairly often)	205	−10.4 (− 17.5, − 3.3)	0.004**	6.8 (1.2, 12.4)	0.017*	3.8 (1.1, 6.4)	0.005**
Signs and symptoms such as pain, ache, nausea, itching, etc. (no signs and symptoms *or* minor signs and symptoms)	195	−18.7 (−29.3, − 8)	0.001**	15 (6.5, 23.4)	0.001**	9.3 (5.3, 13.3)	< 0.001**
Frequency of exercise through swimming (2–3 times a month *or* more)	212	−1.5 (−8.6, 5.5)	0.668	2.3 (−3.2, 8)	0.404	3.6 (0.9. 6.2)	0.008**
Considered retirement before the retirement age (have not *or* have sometimes)	213	3.5 (−5.1, 12.3)	0.424	2.3 (−4.6, 9.2)	0.510	3.4 (0.1, 6.7)	0.044*

The regression coefficients (B) with (95% confidence interval) from generalized linear mixed model controlling for gender and age with urban-rural area as a random effect (except for urban-rural area itself that no random effect was considered). ⁎*p* < 0.05; **p < 0.01

Reference categories: urban-rural areas: inner urban, outer urban, or peri-urban; unable to work due to diseases/injuries: sometimes reduce in work pace or changes to work is needed, often reduce in work pace or changes to work is needed, part time work on account of illness is needed, or totally unable to work; frequency of physical activity through gardening: once a month or less, or not at all; occupational group: directors and senior management, senior advisors and senior officials, advisors and officials, office workers and customer service representatives, and cannot say; difficulty of a 2-km run without breaks: with much difficulty or cannot at all; basic education: matriculation examination; sleeping problems: considerable problems and major problems, or severe insomnia; frequency of exercise through walking: once a month or less, or not at all; total amount of sleep: significantly insufficient or totally insufficient; difficulty of a 5-km run without breaks: with much difficulty or cannot at all; enjoyment of daily activities: now and then, hardly ever, or never; signs and symptoms: considerable signs and symptoms, strong signs and symptoms, or intolerable signs and symptoms; frequency of exercise through swimming: once a month or less, or not at all; considered retirement before the retirement age: have often, or have applied for a pension

Overall, from the regression coefficients (B) in Tables 3 and 4 (indicative of changes in min/day of SB, LPA, and MVPA for every 1 SD change in the predictor and of changes from the reference response categories, respectively), the associations seemed generally stronger for those factors that emerged in the higher layer and larger subgroups. For instance, higher body fat percentage and lower normalized heart rate recovery slope were associated with lower and higher min/day in MVPA, respectively, but the former, which appeared in the higher level of the decision tree, was associated with MVPA to a greater extent (B = -11.7 vs. 9.5).

## Discussion

This study applied the decision tree technique to establish a multilevel data-driven model that predicts adults’ PA behavior, defined as active or inactive based on their machine-learned activity profiles, and to methodologically identify PA behavior correlates. From the 168 factors of different domains used as input variables to create the decision tree model, the final model selected 36 factors from which 54 different participant subgroups with different variations in SED, LPA, and MVPA were formed. The largest subgroup of inactive participants included those with the highest body fat percentage, who were frequently engaged in physically demanding activities through gardening, but who had rather slow heart rate recovery. The largest subgroup of active participants included those with the lowest body fat percentage in the study population with a relatively fast heart rate recovery. The factors that emerged from the decision tree model, such as body fat percentage, normalized heart rate recovery 60 s after exercise, urban–rural areas, average weekday total sitting time, and extravagance score, were associated with SED, LPA, and/or MVPA time. Thus, the present results may inform both multilevel intervention allocation and design.

Consistent with the results of studies focusing on understanding the causation of PA behaviors [5, 8, 13, 44], the established model in the present study indicates that PA behavior is explained by a multilevel hierarchy composed of various factors in different domains. However, our results extend this finding by indicating that PA behavior predictors for different subgroups are different and come from various domains. In addition, our model was driven by empirical data consisting of a range of factors. Studies have generally conceptualized the influence of PA behaviors by theoretically combining common sense and well-established evidence, therefore primarily providing a broad view of PA behavior and its causation for general populations [5, 8, 44]. While previous multilevel models have succeeded in hypothesizing the interaction among factors of different domains, their practical implications have remained limited [8] partially because of their theoretical nature. There were two studies that applied a data-driven approach to establish a decision tree–based model but with self-reported PA measure and a limited number of factors, and one of them used only demographical factors [17] while the other used only sociodemographic factors [16]. Overall, the multilevel model presented here specifies the PA behavior correlates at different levels in each subgroup and may be utilized to target and tailor interventions.

Most emerged factors in the decision tree model have been recognized as factors associated with PA behavior in past works, such as education level, profession, overall health status, fitness status, and population density [5, 6, 11]. However, there were also some factor in decision tree model were less established, such as those that were related to personality and temperament including extravagance, impulsiveness, and explorative excitability [6]. Such factors (or factors similar to them) were assessed in a few studies but, mostly due to the limited or sometimes contradictory evidence, had not yet been identified as correlates nor been rejected. The other factors that can also be categorized as less established factors are body composition measures (i.e., lean body mass and skeletal muscle mass) and a few of the psychological and environmental factors (e.g., enjoyment of daily activities and number of road accidents) [6–8]. A few measures related to heart rate recovery were also emerged in the decision tree model. Even though the association of PA with heart rate recovery measures have been well-studied [45], they can be considered as novel factors associated with PA behavior that are identified in the present study because our results indicate the existence of another direction of relationship that has not been previously examined.

The less established and previously undiscovered factors found here may be candidates for the next generation of correlates [5]. These factors have likely remained underreported (or unexamined) because of the subjective tendency in the existing literature toward examining only those factors for which evidence of significant associations (positive or negative) with different PA behavior indices has been well-established [11]. It is important to consider that these factors were selected by the decision tree to create the final model from a wide list of input (independent) variables. This suggests that the less established and novel factors that emerged in the decision tree model might be relatively more important correlates and likely surrogates for the other previously less established or well-established factors that the decision tree excluded in creating the model, such as behavioral attributes (e.g., alcohol, smoking, etc.) or socioeconomic status [6]. Nevertheless, one must infer the relative importance of the emergent factors with caution. The study’s participants had a narrow age range (46–48 years), which might explain why some of the well-known PA behavior correlates, including age and gender, did not appear in the final model [5, 6, 11, 46]. This result agrees with the findings of a previous review, speculating that in studies including both men and women with sufficient age diversity, age was found to be inversely associated with PA participation, and significant differences in PA participation existed between men and women (higher in men) [11].

As far as we know, our study is the first to use machine-learned activity profiles to define the PA behavior of participants. Previous studies have generally examined the associations between different factors and unidimensional indices, typically including the daily amount of SED, LPA, and/or MVPA [16, 17, 47, 48]. However, recent evidence from time-use epidemiological studies and beyond suggests that these three activities are interrelated [10, 37–39], and should all be considered when studying individuals’ PA behavior [37–39]. Although due to the methodological constraints (i.e., outcome variable imbalance and overlap) we merged all the active participants in one class to form a near-balance and non-overlapping outcome variable [40, 41], it was apparent that the accumulation patterns of SED, LPA, and/or MVPA were varied in the 54 decision-tree formed subgroups and across different active and inactive subgroups. This is indicating that all the three activity intensities along with their interrelationships were considered in our definition of active and inactive individuals, which were based on machine-learned activity profiles [20]. Hence, our multidimensional definition of PA behavior might limit the comparability of our results with those of other studies with unidimensional criteria for defining PA behaviors.

Body fat percentage, a direct measure of adiposity, was the most primary discriminator in the decision tree model. Even though it is typically assumed that PA impacts adiposity-related measures, this result is consistent with the findings of a previous systematic review suggesting a possible bidirectional relationship between adiposity and PA behavior [5]. A number of other factors for which the other direction of relationship is generally assumed were also seen in the other layers of the final model including muscle strength and heart rate recovery measures. Of note is the prognostic value of most of these factors for several chronic health conditions. For example, attenuated heart rate recovery is associated with an increased risk of diabetes [49], or can even indicate the presence of coronary artery disease [50]. Chronic health conditions have been identified both as a barrier and as motivations towards PA in different populations [51]. Even though the self-reported measures addressed the prevalence of diagnosed diseases (e.g., having diabetes, hypertension, etc.), these direct measures were eliminated from the list of input variables due to the high number of missing values. Besides, the study’s participants did not consist of only healthy individuals. As a result, the factors with prognostic value of chronic diseases found in our model may be acting as partial surrogates for chronic health conditions/risks and their effects on different PA behaviors.

We also performed association analysis between all the emerged factors in the decision tree model and three PA metrics. Almost all the emerged factors in the decision tree model were significantly associated with SED, LPA, and/or MVPA. The results of association analyses were, at least for the well-established factors, in line with previous studies. For instance, a better health-related quality of life score was associated with lower levels of SED [52], and higher levels of LPA and MVPA [6]. The results of association analyses also indicated the relative importance of the identified factors, supporting that our results can be used to highlight the factors associating with PA behavior in terms of priority.

The main strength of the present study is the inclusion of a wide list of factors rather than a few subjectively selected factors [5, 11], which resulted in the discovery of the novel predictors. The use of objective measurement of daily PA is also a strength. Previous studies have typically used self-reported PA measures that are known to be imprecise and biased [53]. Another strength is the discrimination of PA behaviors based on activity profiles built using the whole activity intensity spectrum over the course of one full week [10]. However, the binary categorization of participants (active or inactive) might be a limitation. We used a binary outcome variable because the model’s prediction accuracy degraded significantly when the number of PA behavior categories increased (for example, to Inactive, Moderately active and Evening active, and Very active), mostly because of the misclassification between the active categories (results not presented). This is not surprising because despite the different temporal pattern of activities in the active profiles, the overall activity levels were comparable (overlap problem) and the outcome variable was imbalanced. Although it was practically possible to reduce the dimension or select the relevant features in prior to decision tree induction [54], or use more complicated learning algorithms (such as ensemble methods) to achieve a better performance with higher number of categories in the outcome variable [33], these could have caused loss of key mechanistic information and obscured the interpretability of the final model [33, 40, 54], limiting the recognition of novel correlates categories that were identified here. Another limitation is the cross-sectional study design, which prevents any causal effects to be analyzed. Also, although more than 85% of the original cohort members were alive in Finland during the latest follow-up, less than 40% participated and provided valid accelerometer data—possibly those who were healthier and more active. This might induce selection bias and limit the generalizability of the results. Additionally, the study sample was homogenous in terms of age and ethnicity, and some of the emergent factors in the final model were related to cultural and health behaviors. These might also limit the generalizability of the results, especially to more diverse populations with different cultural and health behaviors.

## Conclusion

Using a data mining approach, we established a multilevel model that predicts PA behavior from empirical and large-scale data. The model consisted of 36 different factors of relative importance from different domains and may be used to target and tailor interventions. The factors emerging from the decision tree model such as body fat percentage, normalized heart rate recovery 60 s after exercise, urban-rural areas, average weekday total sitting time, and extravagance score were associated with SED, LPA, and/or MVPA time. The extensive set of factors that was methodologically discovered can be a basis for additional hypothesis testing in PA correlates research. Finally, data mining appeared to be a feasible approach and complex enough to identify different factors along with their interdependencies in explaining PA behavior.

## Supplementary information

**Additional file 1: Table S1.** List of environmental measures. **Table S2.** List of self-reported measures. **Table S3.** List of clinical measures.

## Data Availability

The datasets analyzed in the present study are available from the NFBC Project Centre repository upon request, https://www.oulu.fi/nfbc/node/47960.

## Abbreviations

BMI: Body mass index
CHAID: Chi-squared Automatic Interaction Detection
CI: Confidence interval
LPA: Light physical activity
MET: Metabolic equivalent
MVPA: Moderate-to-vigorous physical activity
NFBC1966: Northern Finland Birth Cohort 1966 study
PA: Physical activity
RI: Response index
S: Subgroup
SED: Sedentary
SD: Standard deviation
TCI: Temperament and Character Inventory
