# Supplementary material for: Correlates of physical activity behavior in adults: a data mining approach
Source: Int J Behav Nutr Phys Act. 2020 Jul 23;17:94. doi: 10.1186/s12966-020-00996-7 (PMC7376928; doi:10.1186/s12966-020-00996-7)
Supplement: Supplementary file 1 — Additional file 1: Table S1. List of environmental measures. Table S2. List of self-reported measures. Table S3. List of clinical measures. [file 12966_2020_996_MOESM1_ESM.docx]

**Table S1**. List of environmental measures (shaded rows are the factors that were emerged in the final decision tree model).

| **Variable** | **Definition** | **Data source** |
| --- | --- | --- |
| **Built environment** |  |  |
| Number of public transportation stops | The number of public transportations stops within a 1-kilometer buffer of residential location. | Finnish Transport Agency: Finnish National Road and Street Database |
| Cycling path length, meters | Cycling paths length within a 1-kilometer buffer of residential location. | Finnish Transport Agency: Finnish National Road and Street Database |
| Distance to local center, meters | The road network distance from residential location to local neighborhood center. | Statistics Finland: Finnish Community Structure data base  Finnish Transport Agency: Finnish National Road and Street Database |
| Distance to closest grocery store, meters | The road network distance from residential location to closest supermarket. | Statistics Finland: Finnish Community Structure data base  Finnish Transport Agency: Finnish National Road and Street Database |
| Number of sport facilities | The number of sport facilities within a 1-kilometer buffer of residential location. | University of Jyväskylä: LIPAS - Finnish database of sport facilities |
| Number of grocery stores | The number of grocery store within a 1-kilometer buffer of residential location. | Statistics Finland: Finnish Community Structure data base |
| Urban-rural area | 1. Inner urban 2. Rural heartland local centers in rural areas 3. Sparsely populated rural areas 4. Peri-urban 5. Rural areas close to urban areas 6. Outer urban | Finnish Environment Institute: Regional classification system |
| Urban zone | 1. Pedestrian zone/sector in regional center. 2. Intensive public transportation in regional center. 3. Public transportation in regional center. 4. Car zone/sector. 5. Weak public transportation. 6. Intensive Public Transportation. 7. Central Pedestrian zone/sector 8. Perimeter of the center/intensive public transportation. 9. Perimeter of the center/public transportation. | Finnish Environment Institute: Regional classification system |
| Population density, per square mile | Density of population within a 1-kilometer buffer of residential location. | Statistics Finland: Finnish Community Structure data base |
| Number of destinations | Number of amenities (sum of destinations for retail, recreation, office and community institutions) within a 1-kilometer buffer of residential location | Statistics Finland: Finnish Community Structure data base |
| Number of intersections | Number of intersections within a 1-kilometer buffer of residential location. | Finnish Transport Agency: Finnish National Road and Street Database |
| Neighborhood walkability | Sum of standardized values of population density, number of destinations and number of intersections within a 1-kilometer buffer of residential location. | Statistics Finland: Finnish Community Structure data base  Finnish Transport Agency: Finnish National Road and Street Database |
| Snow blowing zone | Indicating how often the snow is cleaned from the streets   1. Maintenance class 1 (up to 3 cm removed within 4 hours since snowfall) 2. Maintenance class 2 (up to 6 cm removed within 4 hours since snowfall) 3. Maintenance class 3 (up to 6 cm removed within 12 hours since snowfall) 4. No maintenance | City of Oulu |
| **Natural environment** |  |  |
| Distance to closest forest, meters | The distance from residential location to the closest forest. | Finnish Environment Institute: Land Cover  Finnish Transport Agency: Finnish National Road and Street Database |
| Distance to closest trail, meters | The distance from residential location to the closest trail. | University of Jyväskylä: LIPAS - Finnish database of sport facilities  Finnish Transport Agency: Finnish National Road and Street Database |
| Distance to park, meters | The distance from residential location to the closest park. | Finnish Environment Institute: Land Cover  Finnish Transport Agency: Finnish National Road and Street Database |
| Normalized difference vegetation index (NDVI or greenness index) | A marker of surrounding greenness | Finnish Environment Institute |
| **Socioeconomic environment** |  |  |
| Economic dependency ratio | Number of persons unemployed or outside the labour force per one employed person. | Statistics Finland |
| Number of road accidents | Number of accidents within 1-kilometer buffer of residential environment. | Statistics Finland: Road accidents data base |
| Number of workplaces | The number of workplaces calculated using number of employees whose workplace is located within a 1-kilometer buffer of residential location. | Statistics Finland: Finnish Community Structure data base |
| Number of residential dwellings | Number of residential dwellings within a 1-kilometer buffer of residential location. | Statistics Finland: Finnish Community Structure data base |
| Number of permanently inhabited dwellings | Number of permanently inhabited dwellings within a 1-kilometer buffer of residential location. | Statistics Finland: Finnish Community Structure data base |
| Number of detached houses | Number of detached houses within a 1-kilometer buffer of residential location. | Statistics Finland: Finnish Community Structure data base |
| Number of housing unit in row houses | Number of housing unit in row houses within a 1-kilometer buffer of residential location. | Statistics Finland: Finnish Community Structure data base |
| Number of apartments in apartment houses | Number of apartments in apartment houses within a 1-kilometer buffer of residential location. | Statistics Finland: Finnish Community Structure data base |
| Number of apartments in other housing types | Number of apartments in other housing types within a 1-kilometer buffer of residential location. | Statistics Finland: Finnish Community Structure data base |
| Number of owner-occupied dwellings | Number of owner-occupied dwellings within a 1-kilometer buffer of residential location. | Statistics Finland: Finnish Community Structure data base |
| Number of rental flats | Number of rental flats within a 1-kilometer buffer of residential location. | Statistics Finland: Finnish Community Structure data base |
| Number of dwellings with unknown ownership | Number of dwellings with unknown ownership within a 1-kilometer buffer of residential location. | Statistics Finland: Finnish Community Structure data base |
| Median income per dwelling unit, Euro per year | Median income per dwelling unit within a 1-kilometer buffer of residential location. | Statistics Finland: Finnish Community Structure data base |
| Number of dwelling units | Number of dwelling units within a 1-kilometer buffer of residential location. | Statistics Finland: Finnish Community Structure data base |
| Number of dwelling units with children under 18 years of age | Number of dwelling units with children under 18 years of age within a 1-kilometer buffer of residential location. | Statistics Finland: Finnish Community Structure data base |
| Number of dwelling units with one car | Number of dwelling units with one car within a 1-kilometer buffer of residential location. | Statistics Finland: Finnish Community Structure data base |
| Number of dwelling units with two or more cars | Number of dwelling units with two or more cars within a 1-kilometer buffer of residential location. | Statistics Finland: Finnish Community Structure data base |
| Number of workforce | Number of workforce (i.e., including both employed and unemployed persons) within a 1-kilometer buffer of residential location. | Statistics Finland: Finnish Community Structure data base |
| Number of employed persons | Number of employed persons within a 1-kilometer buffer of residential location. | Statistics Finland: Finnish Community Structure data base |
| Number of employed persons with basic comprehensive education | Number of employed persons with basic comprehensive education within a 1-kilometer buffer of residential location. | Statistics Finland: Finnish Community Structure data base |
| Number of employed persons with secondary education | Number of employed persons with secondary education within a 1-kilometer buffer of residential location. | Statistics Finland: Finnish Community Structure data base |
| Number of employed persons with higher education | Number of employed persons with higher education within a 1-kilometer buffer of residential location. | Statistics Finland: Finnish Community Structure data base |

**Table S2**. List of self-reported measures (shaded rows are the factors that were emerged in the final decision tree model).

| **Question or score (calculated based on** **responses to multiple items)** | **Response categories or definition** | **Data source** | **Short label, if appeared in the model** |
| --- | --- | --- | --- |
| What is your basic education? | 1. Less than 9 years of comprehensive school 2. Comprehensive school 3. Matriculation examination | Background, lifestyle and health survey | Basic education |
| Your current marital status? | 1. Married 2. Cohabiting 3. Partner in a registered partnership 4. Unmarried 5. Divorced 6. Divorced from a registered partnership 7. Widowed 8. Widowed after a registered partnership | Background, lifestyle and health survey |  |
| Smoking status | 1. Non-smoker 2. Current smoker 3. Former smoker | Background, lifestyle and health survey |  |
| Total amount of alcohol intake, gram per day | Summation of responses to several questions regarding drinking habits | Background, lifestyle and health survey |  |
| Do other people live in your household besides you? | 1. No, I live alone 2. Yes | Background, lifestyle and health survey |  |
| Do you live on a working, agricultural or animal production, farm? | 1. Yes 2. No | Background, lifestyle and health survey |  |
| How often do you exercise in your leisure time?  *Light exercise (no sweating or getting out of breath)?* | 1. Once a month or less 2. 2-3 times a month 3. Once a week 4. 2-3 times a week 5. 4 times a week or more 6. Daily | Background, lifestyle and health survey |  |
| How often do you exercise in your leisure time?  *brisk exercise (you get out of breath and sweat at least mildly)* | 1. Once a month or less 2. 2-3 times a month 3. Once a week 4. 2-3 times a week 5. 4 times a week or more 6. Daily | Background, lifestyle and health survey |  |
| How long do you exercise for at any one time?  *light exercise (no sweating or getting out of breath)* | 1. None 2. Less than 20 minutes 3. 20–39 minutes 4. 40–59 minutes 5. 1–1.5 hours 6. More than 1.5 hours | Background, lifestyle and health survey |  |
| How long do you exercise for at any one time?  *brisk exercise (you get out of breath and sweat at least mildly)* | 1. None 2. Less than 20 minutes 3. 20–39 minutes 4. 40–59 minutes 5. 1–1.5 hours 6. More than 1.5 hours | Background, lifestyle and health survey |  |
| How much do you exercise and strain yourself physically in your leisure time? | 1. In my leisure time, I read, watch television and do chores that do not involve much movement or straining myself physically. 2. In my leisure time, I walk, ride a bike or do other types of exercise at least for four hours a week. This includes walking, fishing and hunting, light gardening, etc. but not commuting. 3. In my leisure time, I do actual fitness training, such as running, jogging, skiing, gymnastics, swimming and ball games, or strenuous gardening or other similar tasks on average at least for two hours a week. 4. In my leisure time, I regularly do competitive training several times a week; running, orienteering, skiing, swimming, ball games, or other strenuous sports activities. | Background, lifestyle and health survey |  |
| How many hours do you sit on average in weekdays?  *At home, watching TV or videos*. | Self-reported in hour and minute | Background, lifestyle and health survey |  |
| How many hours do you sit on average in weekdays?  *At home at the computer*. | Self-reported in hour and minute | Background, lifestyle and health survey | Average weekday computer use time |
| How many hours do you sit on average in weekdays?  *In a vehicle*. | Self-reported in hour and minute | Background, lifestyle and health survey |  |
| How many hours do you sit on average during weekdays?  *During the workday at the office or other such place*? | Self-reported in hour and minute | Background, lifestyle and health survey | Average weekday sitting time at the office or other such place |
| Total sitting time in weekdays, hour per weekday | Sum of the responses to all questions about sitting time. | Background, lifestyle and health survey | Average weekday total sitting time |
| Do you currently use snuff or chewing tobacco? | 1. No 2. Occasionally 3. Yes, regularly | Background, lifestyle and health survey |  |
| What vocational qualifications do you have? | 1. No vocational training 2. Vocational course 3. Vocational school 4. College-level training 5. Degree from a university of applied sciences 6. University or other higher education degree 7. Other 8. Training not finished | Background, lifestyle and health survey |  |
| How satisfied are you with your current situation in life in general? | 1. Very satisfied 2. Somewhat satisfied 3. Somewhat dissatisfied 4. Very dissatisfied | Background, lifestyle and health survey |  |
| How would you estimate your current state of health? | 1. Very good 2. Good 3. Moderate 4. Poor 5. Very poor | Background, lifestyle and health survey |  |
| How much the said problem has bothered you within the last week?  *Difficulty in falling asleep.* | 1. No at all 2. Some 3. Considerably 4. Very much | Background, lifestyle and health survey |  |
| How much the said problem has bothered you within the last week?  *Tension or over-exhaustion.* | 1. No at all 2. Some 3. Considerably 4. Very much | Background, lifestyle and health survey |  |
| how much the said problem has bothered you within the last week?  *A feeling of loneliness.* | 1. No at all 2. Some 3. Considerably 4. Very much | Background, lifestyle and health survey |  |
| How much the said problem has bothered you within the last week?  *Such a strong feeling of restlessness that it has been hard to sit still.* | 1. No at all 2. Some 3. Considerably 4. Very much | Background, lifestyle and health survey |  |
| How much the said problem has bothered you within the last week?  *Lack of sexual interest or pleasure* | 1. No at all 2. Some 3. Considerably 4. Very much | Background, lifestyle and health survey |  |
| How much the said problem has bothered you within the last week?  *Lack of energy or impotency.* | 1. No at all 2. Some 3. Considerably 4. Very much | Background, lifestyle and health survey |  |
| How much the said problem has bothered you within the last week?  *Tremor* | 1. No at all 2. Some 3. Considerably 4. Very much | Background, lifestyle and health survey |  |
| How much the said problem has bothered you within the last week?  *Loss of appetite.* | 1. No at all 2. Some 3. Considerably 4. Very much | Background, lifestyle and health survey |  |
| How much the said problem has bothered you within the last week?  *Dejection.* | 1. No at all 2. Some 3. Considerably 4. Very much | Background, lifestyle and health survey |  |
| How much the said problem has bothered you within the last week?  *Lack of interest.* | 1. No at all 2. Some 3. Considerably 4. Very much | Background, lifestyle and health survey |  |
| How tired do you feel for the first half hour in the morning? | 1. Very tired 2. Somewhat tired 3. Somewhat rested 4. Well-rested | Background, lifestyle and health survey |  |
| The degree of difficulty of said problem with sleeping you have experienced (if any), if you have experienced the problem at least three times a week during the last month.  *Total amount of sleep.* | 1. Sufficient 2. Somewhat sufficient 3. Significantly insufficient 4. Totally insufficient | Background, lifestyle and health survey | Total amount of sleep |
| The degree of difficulty of said problem with sleeping you have experienced (if any), if you have experienced the problem at least three times a week during the last month.  *Falling asleep (the time it takes for you to fall asleep after the lights have been turned off in order to go to sleep).* | 1. No problem 2. Somewhat delayed 3. Significantly delayed 4. Very long delay | Background, lifestyle and health survey |  |
| The degree of difficulty of said problem with sleeping you have experienced (if any), if you have experienced the problem at least three times a week during the last month.  *Waking up at night.* | 1. No problem 2. Slight problem 3. Moderate problem 4. Serious problem | Background, lifestyle and health survey |  |
| The degree of difficulty of said problem with sleeping you have experienced (if any), if you have experienced the problem at least three times a week during the last month.  *Night sleep ends too early in the morning.* | 1. Not at all 2. Slightly earlier 3. Significantly earlier 4. Very much earlier | Background, lifestyle and health survey |  |
| The degree of difficulty of said problem with sleeping you have experienced (if any), if you have experienced the problem at least three times a week during the last month.  *Quality of sleep (regardless of how long you slept).* | 1. Satisfactory 2. Somewhat unsatisfactory 3. Significantly unsatisfactory 4. Totally unsatisfactory | Background, lifestyle and health survey |  |
| Have you ever seriously tried to lose weight? | 1. Never 2. Yes, once 3. Yes, several times | Background, lifestyle and health survey |  |
| How do you feel about your weight? | 1. Significantly overweight 2. Slightly overweight 3. Just the right weight 4. Slightly or significantly underweight | Background, lifestyle and health survey |  |
| Duration of brisk physical activity during leisure time, hour per week | Self-reported in hour and minute per week | Background, lifestyle and health survey |  |
| Duration of light physical activity during leisure time, hour per week | Self-reported in hour and minute per week | Background, lifestyle and health survey |  |
| Do your diseases or injuries cause you problems in your current work? (If you are not currently employed, answer from the perspective of your opportunities to find employment) | 1. No problems/no diseases 2. I can carry out my work, but it causes me symptoms 3. I sometimes need to reduce my work pace or make changes to the way I work 4. I often have to reduce my work pace or make changes to the way I work 5. In my opinion, I should only work part time on account of my illness 6. In my opinion, I am totally unable to work | Economic, work life and resource survey | Unable to work due to diseases/injuries? |
| Occupational group | 1. Directors and senior management 2. Senior advisors and senior officials 3. Advisors and officials 4. Office workers and customer service representatives 5. Service, sales and care staff 6. Farmers and forestry workers 7. Building, repair and manufacturing workers 8. Process and transport workers 9. Other workers 10. Cannot say | Economic, work life and resource survey | Occupational group |
| Have you recently been able to enjoy your normal daily routines? | 1. Often 2. Fairly often 3. Now and then 4. Hardly ever 5. Never | Economic, work life and resource survey | Enjoyment of daily activities |
| Have you thought about retiring early, before the statutory retirement age, due to health or other reasons? | 1. No, I have not 2. I have sometimes 3. I have often 4. I have applied for a pension | Economic, work life and resource survey | Considered retirement before the retirement age? |
| Do the following statements apply to you?  *In uncertain situations, I always look forward to the best.* | 1. Totally 2. A lot 3. Somewhat 4. A little 5. Not at all | Economic, work life and resource survey |  |
| Do the following statements apply to you?  *If something can go wrong, in my case it will* | 1. Totally 2. A lot 3. Somewhat 4. A little 5. Not at all | Economic, work life and resource survey |  |
| Do the following statements apply to you?  *I always have a positive and optimistic outlook on the future* | 1. Totally 2. A lot 3. Somewhat 4. A little 5. Not at all | Economic, work life and resource survey |  |
| Do the following statements apply to you?  *I hardly ever expect things to go the way I would want them to go* | 1. Totally 2. A lot 3. Somewhat 4. A little 5. Not at all | Economic, work life and resource survey |  |
| Do the following statements apply to you?  *I hardly ever expect something good to happen to me* | 1. Totally 2. A lot 3. Somewhat 4. A little 5. Not at all | Economic, work life and resource survey |  |
| Do the following statements apply to you?  *I think that more good things than bad things usually happen to me* | 1. Totally 2. A lot 3. Somewhat 4. A little 5. Not at all | Economic, work life and resource survey |  |
| Employment status | 1. Employed 2. Studying 3. Unemployed 4. Other | Economic, work life and resource survey |  |
| What was the total income of your household last year? (before taxes), Euro per year | Self-reported in Euro per year | Economic, work life and resource survey |  |
| Socioeconomic status | 1. Entrepreneurs 2. Agricultural entrepreneurs 3. Senior officials 4. Officials 5. Employees 6. Students 7. Pensioners 8. Other | Economic, work life and resource survey |  |
| Let us assume that your highest work ability index score is 10 points. How would you rate your current ability to work? ('0' means that you are currently totally unable to work) | 1. 1 (Totally unable to work) 2. 2 3. 3 4. 4 5. 5 6. 6 7. 7 8. 8 9. 9 10. 10 (Best possible to work) | Economic, work life and resource survey |  |
| Have you been active and energetic recently? | 1. All the time 2. Fairly often 3. Now and then 4. Hardly ever 5. Never | Economic, work life and resource survey |  |
| Generally speaking, are you fully prepared to take risks, or do you prefer to avoid taking risks? '0' means “not at all ready to take risks” and '10' means “fully prepared to take risks”. | 1. 1 (Not at all ready to take risks) 2. 2 3. 3 4. 4 5. 5 6. 6 7. 7 8. 8 9. 9 10. 10 (Fully prepared to take risks) | Economic, work life and resource survey |  |
| Health-related quality of life questionnaire (15D) score | A single index measure on a 0-1 scale (1 is no problem at all) that combines the responses to Health-related quality of life questionnaire (15D) into one index. | Quality Of Life Questionnaire (15D©) | Overall health-related quality of life score |
| How often do you engage in the following type of sport/exercise? *walking* | 1. Not at all 2. Once a month or less 3. 2-3 times a month 4. Once a week 5. 2-3 times a week 6. 4 times a week or more | Quality Of Life Questionnaire (15D©) | Frequency of exercise through walking |
| How often do you engage in the following type of sport/exercise? *swimming* | 1. Not at all 2. Once a month or less 3. 2-3 times a month 4. Once a week 5. 2-3 times a week 6. 4 times a week or more | Quality Of Life Questionnaire (15D©) | Frequency of exercise through swimming |
| How often do you engage in the following type of sport/exercise? *cycling* | 1. Not at all 2. Once a month or less 3. 2-3 times a month 4. Once a week 5. 2-3 times a week 6. 4 times a week or more | Quality Of Life Questionnaire (15D©) |  |
| How often do you engage in the following type of sport/exercise? *skiing (cross country)* | 1. Not at all 2. Once a month or less 3. 2-3 times a month 4. Once a week 5. 2-3 times a week 6. 4 times a week or more | Quality Of Life Questionnaire (15D©) |  |
| How often do you engage in the following type of sport/exercise? *running* | 1. Not at all 2. Once a month or less 3. 2-3 times a month 4. Once a week 5. 2-3 times a week 6. 4 times a week or more | Quality Of Life Questionnaire (15D©) |  |
| How often do you engage in the following type of sport/exercise? *working out at the gym* | 1. Not at all 2. Once a month or less 3. 2-3 times a month 4. Once a week 5. 2-3 times a week 6. 4 times a week or more | Quality Of Life Questionnaire (15D©) |  |
| How often do you engage in the following type of sport/exercise? *downhill skiing* | 1. Not at all 2. Once a month or less 3. 2-3 times a month 4. Once a week 5. 2-3 times a week 6. 4 times a week or more | Quality Of Life Questionnaire (15D©) |  |
| How often do you engage in the following type of sport/exercise? *aerobics* | 1. Not at all 2. Once a month or less 3. 2-3 times a month 4. Once a week 5. 2-3 times a week 6. 4 times a week or more | Quality Of Life Questionnaire (15D©) |  |
| How often do you engage in the following type of sport/exercise? *gymnastics* | 1. Not at all 2. Once a month or less 3. 2-3 times a month 4. Once a week 5. 2-3 times a week 6. 4 times a week or more | Quality Of Life Questionnaire (15D©) |  |
| How often do you engage in the following type of sport/exercise? *badminton, volleyball, tennis, squash* | 1. Not at all 2. Once a month or less 3. 2-3 times a month 4. Once a week 5. 2-3 times a week 6. 4 times a week or more | Quality Of Life Questionnaire (15D©) |  |
| How often do you engage in the following type of sport/exercise? *floorball, ice hockey, football, rinkball, basketball* | 1. Not at all 2. Once a month or less 3. 2-3 times a month 4. Once a week 5. 2-3 times a week 6. 4 times a week or more | Quality Of Life Questionnaire (15D©) |  |
| How often do you engage in the following type of sport/exercise? *golf* | 1. Not at all 2. Once a month or less 3. 2-3 times a month 4. Once a week 5. 2-3 times a week 6. 4 times a week or more | Quality Of Life Questionnaire (15D©) |  |
| How often do you engage in the following type of sport/exercise? *Shooting* | 1. Not at all 2. Once a month or less 3. 2-3 times a month 4. Once a week 5. 2-3 times a week 6. 4 times a week or more | Quality Of Life Questionnaire (15D©) |  |
| How often do you engage in the following type of sport/exercise? *motor sports (rallying)* | 1. Not at all 2. Once a month or less 3. 2-3 times a month 4. Once a week 5. 2-3 times a week 6. 4 times a week or more | Quality Of Life Questionnaire (15D©) |  |
| How often do you engage in the following type of sport/exercise? *dancing* | 1. Not at all 2. Once a month or less 3. 2-3 times a month 4. Once a week 5. 2-3 times a week 6. 4 times a week or more | Quality Of Life Questionnaire (15D©) |  |
| How often do you engage in the following type of sport/exercise?  *off-road vehicle driving (snowmobile, quad bike, off-road motorcycle)* | 1. Not at all 2. Once a month or less 3. 2-3 times a month 4. Once a week 5. 2-3 times a week 6. 4 times a week or more | Quality Of Life Questionnaire (15D©) |  |
| How often do you engage in the following type of physically demanding activity?  *Gardening* | 1. Not at all 2. Once a month or less 3. 2-3 times a month 4. Once a week 5. 2-3 times a week 6. 4 times a week or more | Quality Of Life Questionnaire (15D©) | Frequency of physical activity through gardening |
| How often do you engage in the following type of physically demanding activity?  *Hiking, camping* | 1. Not at all 2. Once a month or less 3. 2-3 times a month 4. Once a week 5. 2-3 times a week 6. 4 times a week or more | Quality Of Life Questionnaire (15D©) |  |
| How often do you engage in the following type of physically demanding activity?  *Hunting, fishing* | 1. Not at all 2. Once a month or less 3. 2-3 times a month 4. Once a week 5. 2-3 times a week 6. 4 times a week or more | Quality Of Life Questionnaire (15D©) |  |
| How often do you engage in the following type of physically demanding activity?  *Berry-picking* | 1. Not at all 2. Once a month or less 3. 2-3 times a month 4. Once a week 5. 2-3 times a week 6. 4 times a week or more | Quality Of Life Questionnaire (15D©) |  |
| How do you currently manage with the following achievement and activity?  *Bending forward with straight knees, fingers touching the floor* | 1. Without difficulty 2. With some difficulty 3. With much difficulty 4. Cannot at all | Quality Of Life Questionnaire (15D©) |  |
| How do you currently manage with the following achievement and activity?  *20 squats* | 1. Without difficulty 2. With some difficulty 3. With much difficulty 4. Cannot at all | Quality Of Life Questionnaire (15D©) |  |
| How do you currently manage with the following achievement and activity?  *Bending forward with straight knees, fingers touching the floor* | 1. Without difficulty 2. With some difficulty 3. With much difficulty 4. Cannot at all | Quality Of Life Questionnaire (15D©) |  |
| How do you currently manage with the following achievement and activity?  *Sitting up from a laying down position, with your feet straight and without helping with your hands* | 1. Without difficulty 2. With some difficulty 3. With much difficulty 4. Cannot at all | Quality Of Life Questionnaire (15D©) |  |
| How do you currently manage with the following achievement and activity?  *A two kilometer run without breaks* | 1. Without difficulty 2. With some difficulty 3. With much difficulty 4. Cannot at all | Quality Of Life Questionnaire (15D©) | Difficulty of a 2-kilometer run without breaks |
| How do you currently manage with the following achievement and activity?  *A 5-kilometer run without breaks* | 1. Without difficulty 2. With some difficulty 3. With much difficulty 4. Cannot at all | Quality Of Life Questionnaire (15D©) | Difficulty of a 5-kilometer run without breaks |
| How would you estimate your state of health today?  *Mobility* | 1. I can walk normally (without difficulty) indoors, outdoors and on the stairs 2. I can walk without difficulty indoors, but have minor difficulties walking outdoors and/or on the stairs 3. I can walk indoors without assistance (with or without aids), but have considerable difficulties or need assistance when walking outdoors and/or on the stairs 4. Even indoors, I am only able to walk if I am assisted by someone 5. I am totally unable to move and have to stay in bed | Quality Of Life Questionnaire (15D©) |  |
| How would you estimate your state of health today?  *Vision* | 1. My vision is normal; I can read the newspaper and TV subtitles without difficulties (glasses or no glasses) 2. I have minor difficulties reading the newspaper and TV subtitles (glasses or no glasses) 3. I have significant difficulties reading the newspaper and TV subtitles (glasses or no glasses) 4. I cannot read the newspaper or TV subtitles, glasses or no glasses, but my vision is (would be) good enough for moving around without guidance 5. My vision is (would) not be good enough for moving around without guidance, meaning that I am almost or completely blind | Quality Of Life Questionnaire (15D©) |  |
| How would you estimate your state of health today?  *Hearing* | 1. My hearing is normal; I can hear normal speech well (hearing aid or no hearing aid) 2. I have minor difficulties hearing normal speech 3. I have considerable difficulties hearing normal speech; others have to speak louder than normal for me to hear and have a conversation 4. I have major difficulties hearing even loud speech; I am almost deaf 5. I am completely deaf | Quality Of Life Questionnaire (15D©) |  |
| How would you estimate your state of health today?  *Secretion* | 1. My bladder and bowel function normally and without problems 2. I have minor problems with my bladder and/or bowel, e.g. I have difficulty passing urine, or constipation/diarrhoea 3. I have considerable problems with my bladder and/or bowel, e.g. I occasionally have urinary incontinence problems, severe constipation or diarrhoea 4. I have major problems with my bladder and/or bowel, e.g. I have “accidents” on a regular basis, or I need to have an enema or a catheter 5. I am totally unable to control the passage of urine and/or stool | Quality Of Life Questionnaire (15D©) |  |
| How would you estimate your state of health today?  *Physical activity* | 1. I can perform the usual functions normally (e.g. paid work, studying, household work, leisure time activities) 2. My capacity to perform the usual functions is slightly reduced or I have minor difficulties 3. My capacity to perform the usual functions is significantly reduced, or I have significant difficulties, or I can only perform the functions partially 4. I am only able to perform a small percentage of the usual functions 5. I am totally unable to perform the usual functions | Quality Of Life Questionnaire (15D©) |  |
| How would you estimate your state of health today?  *Energy level* | 1. I feel healthy and vigorous 2. I feel slightly unenergetic, tired and powerless 3. I feel somewhat unenergetic, tired and powerless 4. I feel very unenergetic, tired and powerless, almost “burned-out” 5. I feel extremely unenergetic, tired and powerless, totally “burned-out” | Quality Of Life Questionnaire (15D©) |  |
| How would you estimate your state of health today?  *Signs and symptoms* | 1. I have no signs or symptoms, such as pain, ache, nausea, itching, etc. 2. I have minor signs or symptoms, such as minor pain, ache, nausea, itching, etc. 3. I have considerable signs or symptoms, such as considerable pain, ache, nausea, itching, etc. 4. I have strong signs or symptoms, such as strong pain, ache, nausea, itching, etc. 5. I have intolerable signs or symptoms, such as intolerable pain, ache, nausea, itching, etc. | Quality Of Life Questionnaire (15D©) | Signs and symptoms such as pain, ache, nausea, itching, etc. |
| How would you estimate your state of health today?  *Sleeping* | 1. I sleep normally; I have no problems sleeping 2. I have minor sleeping problems, e.g. difficulties falling asleep or tendency to wake up in the middle of the night 3. I have considerable sleeping problems, e.g. I sleep restlessly, I feel like I am not getting enough sleep 4. I have major sleeping problems, e.g. I have to use sleeping pills often or regularly, I wake up regularly in the middle of the night and/or too early in the mornings 5. I suffer from severe insomnia, e.g. despite a heavy use of sleeping pills, I find it impossible to sleep, I am awake most of the night | Quality Of Life Questionnaire (15D©) | Sleeping problems |
| How often do you search for health information on the internet on average? | 1. Never 2. A few times a year 3. Once a month 4. Once a week 5. More than once a week | Temperament and Character Inventory (TCI) |  |
| Worry/pessimism score | Higher scores reflect relatively more pessimism and lower scores reflect relatively more optimism. | Temperament and Character Inventory (TCI) |  |
| Shyness score | Higher scores reflect relatively more shyness and lower scores reflect relatively more outgoing. | Temperament and Character Inventory (TCI) |  |
| Fatigability score | Higher scores reflect relatively more fatigable and lower scores reflect relatively more energetic. | Temperament and Character Inventory (TCI) |  |
| Persistence score | A tendency to act perseveringly even in the face of difficulties or when tired. | Temperament and Character Inventory (TCI) |  |
| Sentimentality score | Higher scores reflect relatively more sentimental and lower scores reflect relatively more detached. | Temperament and Character Inventory (TCI) |  |
| Attachment score | Higher scores reflect relatively warmer and lower scores reflect relatively colder. | Temperament and Character Inventory (TCI) |  |
| Dependence score | Higher scores reflect relatively more appreciative and lower scores reflect relatively more independent. | Temperament and Character Inventory (TCI) |  |
| Novelty seeking score | A tendency for disorderliness, extravagance, impulsivity, quick temper, and actively avoiding frustration. | Temperament and Character Inventory (TCI) |  |
| Harm avoidance score | A tendency to inhibit or stop behavior in response to signals of aversive stimuli. | Temperament and Character Inventory (TCI) |  |
| Reward dependence score | Sensitivity to social cues and dependence on social rewards. | Temperament and Character Inventory (TCI) |  |
| Extravagance score | Higher scores reflect relatively more extravagant and lower scores reflect relatively thriftier. | Temperament and Character Inventory (TCI) | Extravagance score |
| Fear of uncertainty score | Higher scores reflect relatively more fearful and higher scores reflect relatively more daring. | Temperament and Character Inventory (TCI) | Fear of uncertainty score |
| Explorative excitability score | Higher scores reflect relatively more exploratory and lower scores reflect relatively more reserved. | Temperament and Character Inventory (TCI) | Explorative excitability score |
| Disorderliness score | Higher scores reflect relatively more irritable and lower scores reflect relatively more stoical. | Temperament and Character Inventory (TCI) | Disorderliness score |
| Impulsiveness score | Higher scores reflect relatively more impulsive and lower scores reflect relatively more deliberate. | Temperament and Character Inventory (TCI) | Impulsiveness score |

**Table S3.** List of clinical measures (shaded rows are the factors that were emerged in the final decision tree model).

| **Variable** | **Definition** |
| --- | --- |
| Body fat percentage, % | Estimated by InBody 720 body composition analyzer |
| Lean body mass, kilogram | Estimated by InBody 720 body composition analyzer |
| Lean body mass, kilogram | Estimated by InBody 720 body composition analyzer |
| Weight, kilogram | Measured by InBody 720 body composition analyzer |
| Skeletal muscle mass, kilogram | Estimated by InBody 720 body composition analyzer |
| Body fat mass, kilogram | Estimated by InBody 720 body composition analyzer |
| Visceral fat area, cm^2^ | Estimated by InBody 720 body composition analyzer |
| Fitness score | A score provided by Inbody 720 body composition analyzer indicating body condition from a body composition point of view |
| Body mass index (BMI), kilogram/meters^2^ | Weight (kilogram)/[height (meters)]^2^ |
| Waist circumference, centimeters | - |
| Hip circumference, centimeters | - |
| Waist to hip ratio | Waist circumference/hip circumference |
| Normalized heart rate recovery 60 seconds after exercise, % | Heart rate recovery 60 seconds after exercise (described in row 120) normalized by maximum heart rate during step test (described in row 118) |
| Normalized heart rate recovery 30 seconds after exercise, % | Heart rate recovery 30 seconds after exercise (described in row 119) normalized by maximum heart rate during step test (described in row 118) |
| Heart rate recovery 30 seconds after exercise, beats per minute | Heart rate recovery 30 seconds after a submaximal 4-min single step test with a stepping rate of 23 ascents per minute paced by metronome calculated as follows: peak heart rate – heart rate at 30 seconds after exercise. |
| Heart rate recovery 60 seconds after exercise, beats per minute | Heart rate recovery 60 seconds after a submaximal 4-min single step test with a stepping rate of 23 ascents per minute paced by metronome calculated as follows: peak heart rate – heart rate at 60 seconds after exercise. |
| Heart rate recovery slope, beats per minute/second | The steepest 30-second slope during 60 seconds of recovery after a submaximal 4-min single step test with a stepping rate of 23 ascents per minute paced by metronome. |
| Normalized heart rate recovery slope, % per second | Steepest 30-second slope during the first 60 secconds of recovery after exercise (as described in row 121) normalized by maximum heart rate during step test (as described in row 118) |
| Maximum heart rate during step test, beats per minute | Peak heart rate during a submaximal 4-min single step test with a stepping rate of 23 ascents per minute paced by metronome. |
| Age, year | - |
| Gender | 1. Male 2. Female |
| Duration of back endurance test, seconds | Duration of endurance in a static back muscle strength test (Biering-Sorensen trunk extension test) with maximum duration of 4 minutes. |
| Basal metabolic rate, kilocalorie per day | The number of calories one would burn if stayed in bed all day |
